# Light-Addressable Actuator-Sensor Platform for Monitoring and Manipulation of pH Gradients in Microfluidics: A Case Study with the Enzyme Penicillinase

**DOI:** 10.3390/bios11060171

**Published:** 2021-05-27

**Authors:** Rene Welden, Melanie Jablonski, Christina Wege, Michael Keusgen, Patrick Hermann Wagner, Torsten Wagner, Michael J. Schöning

**Affiliations:** 1 Institute of Nano- and Biotechnologies, Aachen University of Applied Sciences, 52428 Jülich, Germany; welden@fh-aachen.de (R.W.); m.jablonski@fh-aachen.de (M.J.); 2 Laboratory for Soft Matter and Biophysics, KU Leuven, 3001 Leuven, Belgium; patrickhermann.wagner@kuleuven.be; 3 Institute of Pharmaceutical Chemistry, Philipps University Marburg, 35032 Marburg, Germany; michael.keusgen@staff.uni-marburg.de; 4 Institute of Biomaterials and Biomolecular Systems, University of Stuttgart, 70569 Stuttgart, Germany; christina.wege@bio.uni-stuttgart.de; 5 Institute of Biological Information Processing (IBI-3), Forschungszentrum Jülich GmbH, 52425 Jülich, Germany

**Keywords:** light-addressable potentiometric sensor, light-addressable electrode, actuator-sensor system, enzyme kinetics, microfluidics

## Abstract

The feasibility of light-addressed detection and manipulation of pH gradients inside an electrochemical microfluidic cell was studied. Local pH changes, induced by a light-addressable electrode (LAE), were detected using a light-addressable potentiometric sensor (LAPS) with different measurement modes representing an actuator-sensor system. Biosensor functionality was examined depending on locally induced pH gradients with the help of the model enzyme penicillinase, which had been immobilized in the microfluidic channel. The surface morphology of the LAE and enzyme-functionalized LAPS was studied by scanning electron microscopy. Furthermore, the penicillin sensitivity of the LAPS inside the microfluidic channel was determined with regard to the analyte’s pH influence on the enzymatic reaction rate. In a final experiment, the LAE-controlled pH inhibition of the enzyme activity was monitored by the LAPS.

## 1. Introduction

Lab-on-a-chip systems, microfluidic bioreactors and organ-on-chip platforms with integrated sensors and actuators for the monitoring of crucial parameters (e.g., flow rate, temperature and pH) are of great interest to maintain micro-environmental conditions [1,2,3]. On the other hand, inducing perturbations of these parameters leads to new perceptions of such systems, e.g., by changing the extracellular pH during cell culturing [4,5,6,7]. Often, due to geometrical restrictions inside microfluidic channels, the flexible integration of “conventional” sensing devices is not easy to accomplish [1]. The sensor information is mostly obtained at a fixed position, predefined during fabrication of the usually rigid sensor geometries. At the same time, a spatially resolved mapping of the molecular species functionalized areas are defined inside the microstructure. In addition, actuation functionalities should be flexible as well, enabling manipulation of e.g., local pH changes without affecting neighboring elements. Therefore, a precise addressability of both the actuator (here, a light-addressable electrode, LAE) and the sensor (here, a light-addressable potentiometric sensor, LAPS) is required.

LAPS is a semiconductor-based chemical sensor which was first proposed by Hafeman et al., in 1988 [8]. LAPS belongs to the group of field-effect-based electrochemical sensors with an electrolyte-insulator-semiconductor (EIS) structure [9]. LAPS offers (depending on its transducer layer) the spatially resolved monitoring of concentration-dependent surface-potential changes, e.g., induced by (bio)chemical/biological molecules or living cells in the analyte solution. LAPS can be designed as multiwell- and multianalyte-sensor devices and can serve for chemical imaging, where the distribution of the analyte concentration is visualized on its chip surface [10,11,12,13,14]. Moreover, LAPS provides a broad range of possible applications and has been utilized for various (bio)chemical and biotechnological approaches, such as monitoring the metabolic activity of bacteria in fermentation broth [14], for on-sensor cryopreservation of cells [15], multi-ion and penicillin detection [16,17], and DNA sensing [18].

In contrast to LAPS, a LAE exhibits no insulating layer, having a direct charge transfer with the analyte. By spatially resolved illumination, conductive areas inside the semiconducting chip can be defined resulting in, e.g., photoelectrocatalytical water oxidation. Furthermore, the LAE can be used for photoelectrochemical material deposition, adjustment of a pH gradient or cell stimulation [19,20,21]. Advantageously, the LAE offers a high flexibility without the need for patterning complicated electrode arrays [22,23]. 

The combination of LAPS and LAE as a sensor-actuator system would enable the simultaneous, spatially resolved pH manipulation and monitoring inside a microfluidic system. This way, reaction processes taking place inside microchannels could be further analyzed and optimized, which is helpful for e.g., studying the response characteristics of immobilized bioreceptors in a microfluidic channel, such as enzymes. 

The proposed experimental approach of a light-addressable actuator-sensor platform (consisting of a LAE and a LAPS) elaborates for the first time the mutual reaction of local pH gradients inside a microfluidic channel and the enzyme-triggered sensor signal. Penicillinase has been selected as a model enzyme to detect penicillin, since it is a robust enzyme making it advantageous for experimental application. The enzymatic reaction induces a pH change, which can be detected with the LAPS. Si_3_N_4_ was used as pH-sensitive transducer layer for the LAPS, and TiO_2_ as photoelectrocatalytical material inducing pH-value manipulations.

The surface morphology of the LAE and the LAPS surface where physically characterized by means of scanning electron microscopy (SEM). The penicillin sensitivity of the LAPS and the effect of pH changes on the enzyme activity, provoked by the LAE, were evaluated by photocurrent-voltage and chemical-image measurements. Dynamic pH variations induced by the LAE can be further used to control the enzymatic reaction rate and to adjust the biosensor response. 

## 2. Materials and Methods

### 2.1. Fabrication Process of Light-Addressable Electrodes (LAEs)

The LAE employed in this study consists of a glass/SnO_2_:F/TiO_2_ heterostructure. The SnO_2_:F glass substrate (7 Ω∙sq^−^^1^) was purchased from Sigma Aldrich (Darmstadt, Germany). The SnO_2_:F glass substrate was cleaned in an ultrasonic bath with acetone, 2-isopropanol and deionized water for 5 min, respectively, and dried with nitrogen. Afterwards, the TiO_2_ layer was deposited by pulsed laser deposition (PLD). During the PLD process, a TiO_2_ target (MaTeck Material-Technologie and Kristalle GmbH, Jülich, Germany) was vaporized with a KrF-excimer laser (λ = 248 nm) using a power density of 5.0 J∙cm^−^^2^ with a repetition frequency of 10 Hz at a pressure of 2.0 *×* 10^−^^2^ hPa O_2_ for 700 s. Hereby, the SnO_2_:F glass substrate was heated during the PLD process to 400 °C to achieve a rutil crystal structure. To achieve an inlet and outlet for the later prepared microfluidic structure, two holes with a diameter of 1.2 mm were drilled in the LAE.

### 2.2. Preparation of Light-Addressable Potentiometric Sensor (LAPS) Chips

The utilized LAPS chips, consisting of a n-Si/SiO_2_/Si_3_N_4_-multilayer structure, were acquired from SEIREN KST Corp. (Fukui, Japan). The thickness of the n-Si, SiO_2_ and Si_3_N_4_ layer was 100 µm, 50 nm and 50 nm, respectively. To remove the surface-oxide layer, the rear side was treated by wet-chemical etching with hydrofluoric acid (HF). Afterwards, a 300 nm thick aluminum (Al) film was deposited by electron-beam evaporation with a deposition rate of 2 nm∙s^−1^ to contact the n-Si substrate electrically. The wafer was diced into 20 × 20 mm^2^ chips and an optical window (ca. 15 × 15 mm^2^) was made by etching an inner rectangle of the Al layer with 5% HF, leaving an outer Al frame.

### 2.3. Enzyme Immoblization with Tobacco Mosaic Virus Particles as Enzyme Nanocarriers on LAPS Chips

For enzyme immobilization, *tobacco mosaic virus* (TMV) particles were utilized as enzyme nanocarriers. A TMV variant (S3C) that exposes a cystein residue on each coat protein was used [24,25]. To functionalize the TMV particles, bifunctional biotin-linker molecules (EZ-Link Maleimide-PEG11-Biotin, ThermoScientific, Rockford, IL, USA) were covalently bound to the thiol groups located on the surface of each coat protein, as described in [26,27,28]. The biotinylated TMV particles were suspended in 10 mM sodium-potassium-phosphate (SPP) buffer (pH 7.0) and stored at 4 °C until use. As a model enzyme, penicillinase from *Bacillus cereus* (Sigma-Aldrich, Darmstadt, Germany) was utilized. For specific enzyme immobilization to the biotin linkers on the surface of the TMV nanotubes, the enzyme was conjugated with streptavidin molecules using a commercial streptavidin conjugation kit (LNK162STR, Bio-Rad, Feldkirchen, Germany) [26,27]. The streptavidin-conjugated penicillinase ([SA]-penicillinase) was stored in 10 mM phosphate-buffered saline (PBS) buffer (1000 Units∙mL^−^^1^, pH 7.0) at 4 °C until further use.

The LAPS chips were cleaned in an ultrasonic bath for 5 min in acetone, 2-isopropanol, ethanol and deionized water, respectively. After drying with nitrogen, 10 µL TMV solution (0.1 mg∙mL^−^^1^) were drop-coated on the Si_3_N_4_ surface of the later-on microfluidic channel, and incubated for 1 h at room temperature (RT) in a humid chamber. Afterwards, the solution with unbound TMV was washed away with deionized water and the sensor chip was dried with nitrogen. In the next step, 5 µL [SA]-penicillinase solution were drop-coated on the immobilized TMV particles and incubated for 1.5 h at RT in a humid chamber. After the incubation time, the sensor chip was rinsed again with deionized water to remove unbound enzyme molecules and dried with nitrogen.

### 2.4. LAPS-LAE Microfluidic Assembly

**Figure 1:**
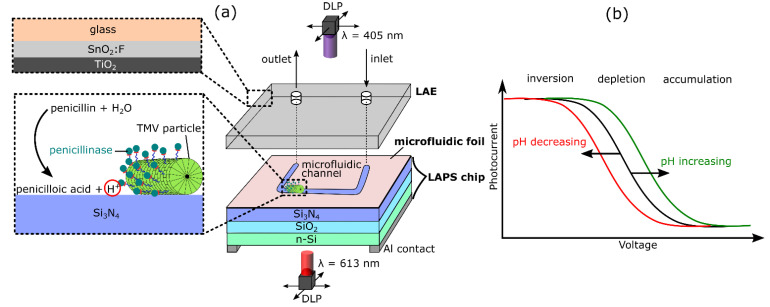
(**a**) Schematic of the microfluidic setup with a light-addressable potentiometric sensor (LAPS)/microfluidic foil/ light-addressable electrode (LAE)-sandwich structure. *Tobacco mosaic virus* (TMV) particles functionalized with the enzyme penicillinase are immobilized inside the microchannel. (**b**) Typical shape of a photocurrent-voltage curve for a n-type LAPS with characteristic regions of inversion, depletion and accumulation.

To prepare the LAPS/microfluidic foil/LAE sandwich structure (schematically depicted in Figure 1a, a ~86 µm thick double-sided adhesive microfluidic tape (3M^TM^, St. Paul, MN, USA) was patterned by laser cutting using a ProtoLaser U3 (LPKS Laser and Electronics AG, Garbsen, Germany). A 20 *×* 20 mm^2^ rectangle with a 1.0 mm wide channel was cut out of the tape. The LAE was cleaned in an ultrasonic bath with acetone, 2-isopropanol, ethanol and deionized water and finally dried with nitrogen. Afterwards, the laser-cut microfluidic foil was stuck onto the TiO_2_ surface of the LAE, positioning the drilled holes of the LAE at the inlet and outlet of the microfluidic channel. In the final step, the TMV- and penicillinase-functionalized LAPS chip was placed below the LAE-microfluidic structure, immobilizing the enzyme-loaded TMV particles at the bottom of the microfluidic channel. For tube connection, ferrules were attached to the inlet and outlet with the help of a photopolymer.

### 2.5. Measurement Setup and Characterization Methods

The inlet tube of the microfluidic setup was connected to a syringe-driven pump system (neMESYS 290N, Cetoni GmbH, Korbussen, Germany) to control the flow inside the channel. In the outlet tube, a Pt-counter electrode and a reference electrode (DRIREF-2SH, World Precision Instruments, Sarasota, FL, USA) for the electrical connection of the LAE and LAPS were inserted. A clamp connected the Al rear side contact of the LAPS to a transimpedance amplifier (gain = 10^7^ V∙A^−1^, AMP100, Thorlabs GmbH, Bergkirchen, Germany) to convert the alternating photocurrent into a measurable voltage. The voltage was recorded by a measurement card (USB 7855R, NI, Austin, TX, USA). The same card also provided the bias voltage to the LAPS with respect to the counter electrode. A potential was directly applied to the LAE and Pt-counter electrode by a source measurement unit (2600b, Keithley Instruments, Solon, OH, USA). 

The LAE and LAPS rear side were illuminated by a digital light processing (DLP) projector (STAR-07, ViALUX Messtechnik + Bildverarbeitung GmbH, Chemnitz, Germany) with a 405 nm and a 613 nm light-emitting diode (LED) light source. Both DLPs are modified with a lens system to focus each of the 1024 × 768 micromirrors to a size of 10 × 10 µm^2^. All measurements were performed in a dark Faraday cage at room temperature (RT). For all experiments, 0.33 mM PBS buffer was used. The pH was adjusted by titration with NaOH and HCl. For penicillin detection, varying concentrations of penicillin G (Sigma Aldrich, Darmstadt, Germany) were added to the measurement solution. 

For photoelectrocatalytically induced pH changes, a constant potential of 300 mV was applied to the LAE with respect to the Pt-counter electrode. After reaching steady- state conditions, the rear side was illuminated with spots of various sizes. 

For LAPS characterization, three measurement modes were applied: photocurrent-voltage (I-V), chemical-image and photocurrent-time mode. The illumination of the LAPS-DLP projector was modulated with a frequency of 512 Hz to achieve an alternating photocurrent for all measurements. The voltage from the transimpedance amplifier was sampled with a frequency of 50 kHz and further processed. For I-V curves, the measurement time for each bias voltage was 400 ms. Figure 1b depicts a theoretical I-V curve of a n-type silicon LAPS. In the I-V mode, the applied voltage was swept from 0 to −3.0 V while measuring the photocurrent for a fixed illumination spot. The typical output curve shows the three characteristic regions of inversion, depletion and accumulation. A pH increase or decrease shifts the I-V curve to more positive or negative voltage, respectively, which is particularly evident in the depletion region. As slight changes of the photocurrent amplitude can occur when replacing the measurement solutions, the I-V curves were normalized with respect to the inversion region. 

To obtain spatially resolved images from the microfluidic channel, the chemical image mode was utilized. A constant bias voltage, chosen close to the inflection point of the I-V curve, was applied and the rear side was scanned sequentially with a moving light beam and a sampling time of 200 ms for each spot (250 × 250 µm^2^). In the results section, differential chemical images are visualized. For that, the chemical image after the enzymatic reaction was subtracted from the initial reference chemical image (before enzymatic catalysis of penicillin by penicillinase). On the basis of the exemplary I-V curve in Figure 1b, at a fixed bias voltage, a pH decrease results in a photocurrent drop, while it increases with rising pH values. 

Similarly, during the photocurrent-time mode, the temporal change of the photocurrent is measured for a fixed illumination spot and a fixed bias voltage (from the I-V inflection point), enabling the dynamic detection of pH changes. The sampling time was 1 s. 

With all three measurement modes, changes of the pH value in the solution can be monitored. In this work, the resulting pH changes are caused by two different proton generation mechanisms, being photoelectrocatalysis and enzymatic conversion. The induced pH change from the LAE originates mostly from the water oxidation reaction,

2 H_2_O → 4 e^−^ + 4 H^+^ + O_2_, (1)

where water is split into H^+^ ions and oxygen. The second pH change is due to the immobilized enzyme penicillinase, where penicillin is converted to penicilloic acid and H^+^ ions through the enzyme’s ß-lactamase activity,

penicillin + H_2_O → penicilloic acid + H^+^. (2)

## 3. Results

### 3.1. Scanning Electron Microscopy (SEM) Characterization of the TiO_2_- and Tobacco Mosaic Virus (TMV)-Modified Si_3_N_4_ Surface

To characterize the surface morphology of the fabricated TiO_2_ and the enzyme-modified Si_3_N_4_, scanning electron microscopy (SEM) images were taken with a Schottky field-emission microscope (JSM-7800F, JEOL GmbH, Freising, Germany). For higher conductivity, a ~5 nm platinum-palladium layer was sputtered onto the Si_3_N_4_ surface before SEM images were taken. 

**Figure 2:**
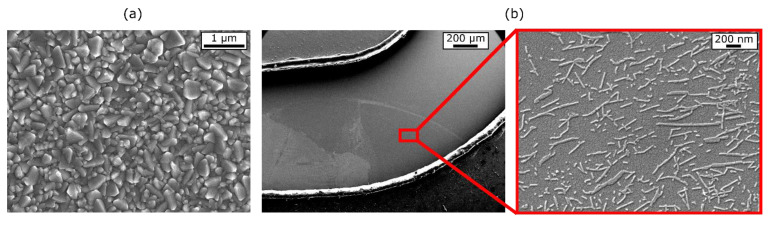
(**a**) Scanning electron microscope (SEM) image (magnification of 20,000× of the LAE showing the TiO_2_ film surface on the SnO_2_:F glass substrate. (**b**) SEM image depicting the part of the microfluidic channel where the enzyme-modified Si_3_N_4_ surface of the LAPS is located with a magnification of 60× (left) and with a zoom-in, showing the adsorbed TMV particles carrying the immobilized penicillinase with a magnification of 35,000× (right).

To achieve a high spatial resolution of the LAE, a low current in the absence of illumination is required. This can be achieved with a dense and non-porous TiO_2_ layer to avoid short circuits with the SnO_2_:F glass [29]. Additionally, it is important to exclude a direct contact of the analyte with the highly-doped SnO_2_:F layer, to circumvent unexpected surface reactions. A representative SEM image of the TiO_2_ surface is given in Figure 2a. The image shows a homogeneous and dense surface structure without visible cracks. The granularity (200–250 nm) is induced by the SnO_2_:F glass on which the ~200 nm TiO_2_ layer is deposited. 

Exemplary SEM images of the TMV-modified (carrying the penicillinase molecules) LAPS-Si_3_N_4_ surface with part of the microfluidic channel are shown in Figure 2b (left and right). The channel boundary is cleanly cut with no visible fringes. On the Si_3_N_4_, the white cloud-like area is indicating the TMV-modified surface spot (left image). A zoom into this spot (right image) shows homogeneously distributed TMV particles with immobilized penicillinase. The TMV particles appear as typical 300 nm long nanotubes, as well as in shorter particle fractions (~50–200 nm) or elongated “end-to-end”—multimer structures (up to ~600 nm) as described in previous works as enzyme nanocarriers on a Ta_2_O_5_-sensor surface [24,26]. 

### 3.2. Penicillin Detection with Penicillinase-Modified LAPS

**Figure 3:**
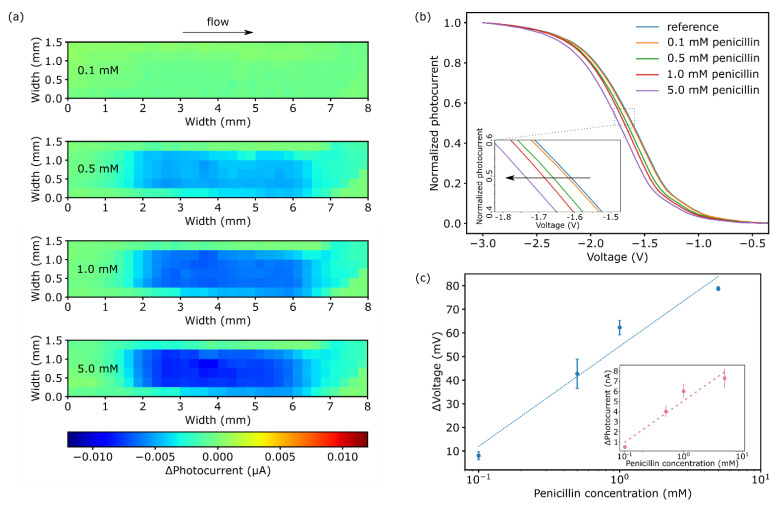
(**a**) Chemical images and (**b**) photocurrent-voltage curves for penicillin concentrations ranging from 0.1 to 5.0 mM after 5 min of enzymatic reaction in phosphate buffered saline (PBS) buffer, pH 7.0. (**c**) Mean calibration curve evaluated from the photocurrent-voltage curves (*n* = 4) with an average penicillin sensitivity of 42.3 mV/dec. The inlet represents the photocurrent change in dependence of the penicillin concentration, evaluated from Figure 3a.

TMV particles were loaded inside the microchannel by drop-coating with subsequent penicillinase coupling by affinity binding of SA-penicillinase conjugates to the biotinylated TMV. Because modified TMV particles have been utilized for the first time inside a microfluidic channel for enzyme immobilization, chemical images and photocurrent-voltage curves were recorded by the LAPS to control the layout’s functionality for penicillin detection. During the enzymatic conversion of penicillin to penicilloic acid, H^+^ ions are generated resulting in a local pH change in the solution. As a first experiment, the pH change resulting from varying penicillin concentrations was studied as chemical images inside the microchannel. The rear side of the LAPS is therefore scanned sequentially, with the resulting photocurrent depicted in Figure 3a. Each chemical image represents (from top to bottom) a different penicillin concentration (from 0.1 mM to 5.0 mM) as differential image. This differential image is obtained by subtracting the particular chemical image from the reference chemical image of the microfluidic structure recorded at an applied potential of −1.65 V in 0.33 mM PBS buffer at pH 7.0. For the reference, (Figure S1 (Supplementary Information)), a high flow rate of the analyte of 1.0 µL∙s^−1^ was chosen to suppress any pH changes inside the channel. During the enzymatic experiments, the flow was stopped, allowing an accumulation of enzymatically produced H^+^ ions. The resulting differential chemical images (Figure 3a) show a section of the microfluidic channel with 192 measurement points, visualizing a total area of 8.0 × 1.5 mm^2^. The applied potential of −1.65 V was selected to be close to the inflection point of the photocurrent–voltage curve (Figure 3b).

In Figure 3a, the bottom image shows the result for a penicillin concentration of 5 mM. Due to the H^+^ ion generation, the ΔPhotocurrent (ΔI_photo_) decreased in the area with immobilized enzyme. The diameter of the pH-change region (~4.0 mm), corresponds to the drop-coated area of the TMV particles with immobilized penicillinase. In this area, ΔI_photo_ changed (decreased) by 7.3 ± 0.9 nA for a penicillin concentration of 5.0 mM. For lower penicillin concentrations, the ΔI_photo_ variations have been 6.0 ± 0.7 nA (1.0 mM) and 4.0 ± 0.5 nA (0.5 mM). For the lowest penicillin concentration (0.1 mM), a small photocurrent (ΔI_photo_ = 0.5 ± 0.0 nA) was detected. Here, due to the ΔI_photo_ scaling of the depicted chemical image, the change is hardly visible. Interestingly, the spatial pH change expansion for all concentrations was—more or less—in the same local area at 4.0 mm width (*x*-axis) and equally distributed indicating a rather low H^+^ ion diffusion away from the enzyme into the surrounding medium; only the photocurrent intensity changed by varying penicillin concentrations. Besides offering a mapping of the enzyme activity inside the microchannel, the chemical image mode can be used to determine the exact spots of immobilized enzymes. Moreover, possible enzyme detachment (e.g., due to shear stress induced by high flow rates) would be directly recognized. 

In addition to the chemical images, for photocurrent–voltage curves, the photocurrent is recorded at a defined location inside the microchannel, while sweeping the applied bias potential from 0 to −3.0 V. The measurement spot with an illumination size of 250 × 250 µm^2^ was located in the center of the determined pH-change area (x-axis 4.0 mm, y-axis 0.75 mm). Figure 3b displays the normalized I-V curves related to the previously discussed chemical images for different penicillin concentrations. The I-V curves exhibit the characteristic regions with inversion, depletion and accumulation. For example, the blue I-V curve represents the previously described reference measurement during vigorous flushing of the channel with PBS buffer solution (1.0 µL∙s^−1^). In the diagram, from −3.0 V to −2.4 V, the n-type semiconductor is in the inversion state. In the depletion region, the photocurrent decreases until an applied voltage of −1.0 V and reaches its minimum due to charge accumulation for further increasing bias potentials. During the enzymatic reaction, the H^+^ ion generation leads to a surface protonation of the Si_3_N_4_, followed by a shift of the photocurrent-voltage curve to more negative potentials. The potential changes were taken from the inflection point at the normalized photocurrent of 0.5. In contrast to the chemical image, a potential change (ΔU) of 8.0 ± 1.7 mV with respect to the reference I-V curve was detected even for 0.1 mM penicillin. For higher penicillin concentrations, the signal shift increased to 42.8 ± 6.2 mV, 62.3 ± 3.1 mV and 78.8 ± 0.8 mV for 0.5 mM, 1.0 mM and 5.0 mM, respectively. The evaluated calibration curve is depicted in Figure 3c. A mean penicillin sensitivity in the concentration range from 0.1 to 5.0 mM of 42.3 mV/dec was achieved. 

The experiments highlight, that the combination of LAPS and penicillinase-functionalized TMV can be used for the detection of penicillin inside a microfluidic channel: here, a two-dimensional mapping in x- and y-directions is possible. 

### 3.3. Impact of pH Changes on Penicillinase Activity

**Figure 4:**
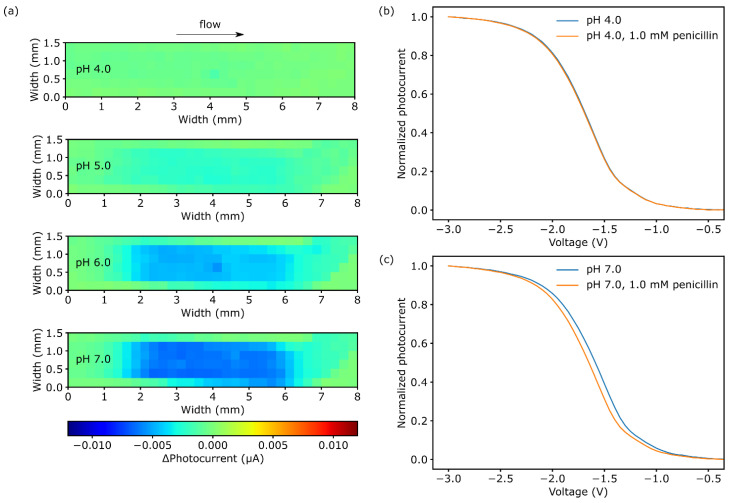
(**a**) Chemical images for 1.0 mM penicillin in pH 4 to pH 8 PBS buffer after 5 min of enzymatic reaction. Photocurrent-voltage curve for (**b**) pH 4 and (**c**) pH 8 PBS buffer with and without 1.0 mM penicillin.

A main aim of this study is to control the rate of enzymatic conversion by locally induced pH changes with the LAE. Therefore, the activity of penicillinase for pH values ranging from pH 4.0 to pH 7.0 in 0.33 mM PBS buffer with a constant penicillin concentration of 1.0 mM was characterized. The differential chemical images in Figure 4a visualize typical pH changes due to H^+^ ion accumulation after stopping the enhanced flow of 1.0 µL∙s^−1^. All results were obtained 5 min after the flow stopped. For pH 4.0, there is nearly no pH change, and thus no change in photocurrent (ΔI_photo_ = 0.4 nA ± 0.0) detected, which can be attributed to the inhibition of the enzyme at a such low pH value (see also activity behavior of penicillinase [30]. For pH 5.0, a slight variation in photocurrent of 2.1 ± 0.1 nA occurs, indicating a low enzymatic activity. For higher pH values of pH 6.0 and pH 7.0, ΔI_photo_ is increased to 4.3 ± 0.4 nA and 5.9 ± 0.6 nA, respectively. Here, the pH values are closer to the penicillase’s activity optimum of approximately pH 7.5 (for immobilized enzyme) [31], resulting in a higher catalytic conversion of penicillin.

These results are confirmed by the associated photocurrent-voltage (I-V) curves, recorded in the center of the area with immobilized enzyme (x-axis 4.0 mm, y-axis 0.75 mm). The corresponding I-V curve for pH 4.0 PBS buffer solution is shown in Figure 4b. Between the reference (blue) and 1.0 mM penicillin I-V curve (orange), there is only a marginal shift of 6.8 ± 3.6 mV. Based on the original pH sensitivity of the sensor of 40 mV∙pH^−1^ (data not shown), this refers to an additional pH drop of 0.17. For pH 7.0 (Figure 4c), the I-V curve shifted by 61.0 ± 0.7 mV, which is equivalent to a pH change of 1.5 from pH 7.0 to pH 5.5. Lowering the pH values in relation to pH 7.0, the photocurrent–voltage curves shifted to more negative voltages by 49.8 ± 2.8 mV at pH 6.0 and 27.0 ± 3.0 mV for pH 5.0 (not shown). It should be mentioned that, beside the inhibited enzyme’s activity at lower pH values, the PBS buffer capacity is also reduced at lower pH values. By that, the enzymatically produced H^+^ ions at pH 5 and pH 4 have a higher impact on the resulting pH change, than at higher pH values of pH 7 and pH 6.

Both, the chemical images and the I-V curve reveal the possibility to inhibit the enzymatic reaction by lowering the pH in a microfluidic setup, where the chemical imaging allows determination of 2-dimensional distribution of pH-triggered enzyme activity.

### 3.4. pH Manipulation with LAE

**Figure 5:**
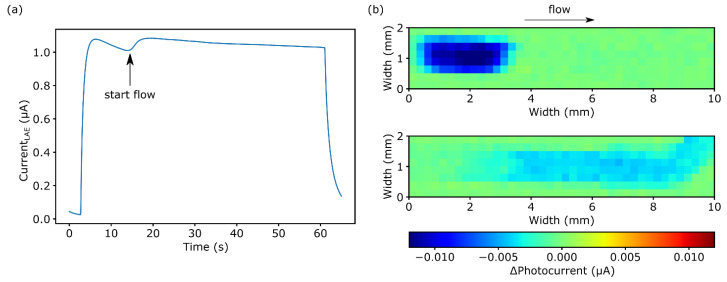
(**a**) Transient photocurrent signal for 60 s of illumination. (**b**) Chemical images of static (top) and dynamic (bottom) pH changes inside the microfluidic channel induced by the LAE.

By utilizing a LAE, a direct charge transfer at the semiconductor/electrolyte interface between generated holes and species in the solution is possible. Depending on the applied LAE potential, e.g., photoelectrocatalytic water splitting takes place, allowing a flexible pH-value adjustment inside the channel. First, a potential of 0.3 V is applied to the LAE against the Pt-counter electrode. A typical transient current response is rendered in Figure 5a. Without illumination, the current equilibrates at 18 nA (3 s). During this condition, no surface reactions are triggered. When illuminating the rear side of the LAE with an area of 0.25 × 1.0 mm^2^, a current peak occurs (4–5 s), which can be assigned to accumulated holes perturbing the surface, resulting in a capacitive discharge [32]. Afterwards, the current equilibrates at 1.04 µA after 50–60 s. Most of the current occurring during illumination can be assigned to the photoelectrocatalytic oxygen-evolution reaction of water where, besides oxygen, H^+^ ions are produced, resulting in a pH change. After switching-off the illumination, the current decreases again to its dark current value.

Similarly to the H^+^ ions generated by the enzymatic reaction, it is possible to visualize the photoelectrocatalytically produced protons with differential chemical images by the LAPS. In Figure 5b, differential chemical images of a 2.0 × 10.0 mm^2^ area and an applied potential of −1.45 V were recorded in PBS buffer, pH 7.1. The top image shows a pH change induced by the LAE without flow. It can be seen that in the illuminated area, the LAPS photocurrent changes by 10.7 ± 1.9 nA. The corresponding I-V curve reveals a shift of 134.4 mV to more negative voltages, which is equal to a pH decrease by ~3.5. 

To exclude that the LAE illumination wavelength of 405 nm affected the functionality of the enzyme, the LAE illumination spot was positioned 3–4 mm downwards (in flow direction) from the enzyme. After 10 s of photoelectrocatalysis, a steady flow of 0.05 µL∙s^−1^ was applied, moving the generated protons upwards the channel to the location of the immobilized enzyme. Hereby, an equilibrium between the proton generation and transport is formed, resulting in a consistent pH change inside the channel. The result is depicted in the bottom image in Figure 5b. The differential chemical image shows an equally distributed pH variation with an average change of the LAPS photocurrent of 3.4 ± 0.6 nA. From the related I-V curve, a pH decrease of 1.0 (ΔU = 41.2 mV) was obtained. Additionally, the start of the flow can be seen in the transient current measurement in Figure 5a. After 10 s of illumination, there is a small current increase. Since the volume above the illuminated area of 1.0 × 0.25 mm^2^ is only 0.02 µL, due to the fluidic dimensions and no-flow conditions during the first 10 s, a reduced mass transfer can lead to a depletion of reaction partners, decreasing the current [33]. Hence, providing fresh solution with starting the flow after 10 s, the current increases to a nearly constant value of 1.04 µA, as the reaction rate between generated electron-hole pairs and reaction partners in solution equilibrate. 

### 3.5. Regulation of Enzyme Activity by the LAE

In the final experiment, the enzymatic activity of penicillinase was directly regulated by the LAE inside the microfluidic channel. Here, photocurrent-time measurements (called as constant potential LAPS measurements) were performed to study the temporal photocurrent change during H^+^ ion generation of the enzymatic reaction. The applied potential was −1.45 V.

**Figure 6:**
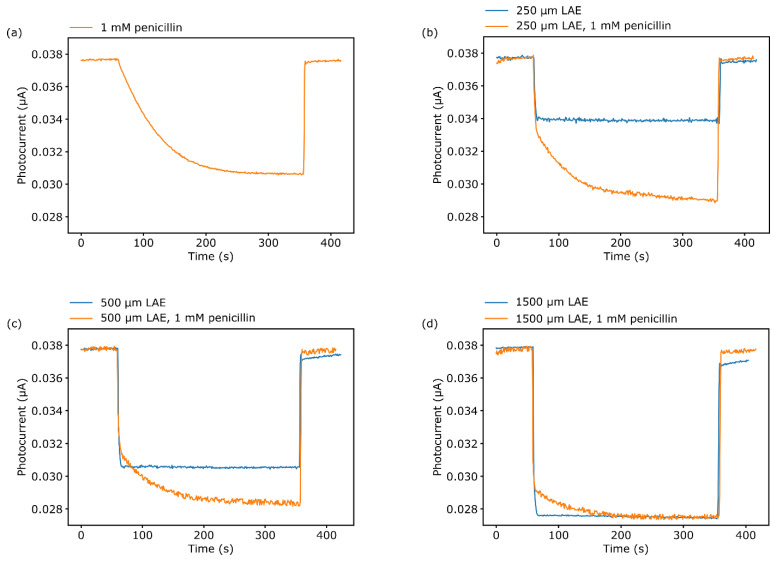
Constant potential LAPS measurements. (**a**) Photocurrent response for 1.0 mM penicillin in PBS buffer, pH 7.1. In (**b**–**d**) the blue curves depict the transient photocurrent decrease due to pH changes induced by the LAE with an illumination width of 250 µm, 500 µm and 1500 µm, respectively. The orange curves show the concatenated, additional change in photocurrent when 1.0 mM penicillin is added to the PBS buffer.

Figure 6a shows the photocurrent change for 1.0 mM penicillin in PBS buffer, pH 7.1, without manipulation of the pH with the LAE. The microchannel was rinsed with a pump rate of 1.0 µL∙s^−1^ for the first 60 s. After stopping the dosage, the photocurrent starts to decrease due to the accumulation of enzymatically produced H^+^ ions and reaches an equilibrium after around 300 s. The measured photocurrent drop of 7.0 nA corresponds to a pH change of 1.75. After 300 s, the flow was started again and the channel was rinsed with fresh solution, whereby the photocurrent increased again to its initial value. 

In the following measurements, the pH was regulated by the LAE and the enzymatic response was determined by the LAPS. First, the LAE induced a pH change by photoelectrocatalytic water oxidation in PBS buffer without penicillin. Since a simultaneous operation between LAPS and LAE is not possible, due to the influence of the 405 nm light beam of the DLP projector on the LAPS chip during measurements, the illustrated curves in Figure 6b–d are concatenated and normalized: The photocurrent was measured during the 60 s of rinsing, stopped while the LAE pH changed, and directly started after the LAE illumination was switched off. During the LAE illumination, the output flow of the pump was reduced to 0.05 µL∙s^−1^. In Figure S2 (Supplementary Information), the effect of the lower flow velocity on the enzyme activity without LAE is analyzed. After changing the flow rate from 1.0 µL∙s^−1^ to 0.05 µL∙s^−1^, a small drop of ΔI_photo_ = 0.5 nA occurred. Nevertheless, the delta photocurrent after stopping the flow reached again 7.0 nA, which is identical to the photocurrent change without the decreased flow rate.

As the change of the pH is defined by the number of generated H^+^ ions during the photoelectrocatalytical water oxidation, this can be influenced by varying the reactive area of the LAE with differently sized illumination spots. These have been changed with illumination lengths inside the microchannel between 250 µm and 1500 µm with the help of the DLP projector.

The pH change for an illuminated area of 1.0 × 0.25 mm^2^ is depicted in Figure 6b. The blue curve shows the constant photocurrent shift of 3.8 nA that corresponds to a pH shift from pH 7.1 to pH 6.3. Subsequently, the measurement was repeated with 1.0 mM penicillin in the PBS buffer (orange curve). Here, after the LAE-induced pH drop, the photocurrent further decreased until it reached an equilibrium after approximately 300 s. The total photocurrent change is 8.6 nA. This is equivalent to a pH change of 2.1. As the LAE altered the pH value to 6.3, the additional pH drop by the enzymatic reaction was 1.3. In Figure 6c, the LAE was illuminated with a beam width of 500 µm, leading to a photocurrent drop of 7.2 nA. With 1.0 mM penicillin, it changed by 9.4 nA. The pH therefore decreases from pH 7.1 to pH 5.4 and pH 4.8 after the LAE and penicillin reaction, respectively. The largest illumination width of 1500 µm resulted in a ΔI_photo_ of 10.3 nA, which corresponds to a pH change of 2.6 (Figure 6d). Since this change already results in pH 4.4 inside the microchannel, no further change in the photocurrent was observed while adding 1.0 mM penicillin in the solution. This indicates that the enzymatic catalysis of penicillin was inhibited. These results underline the high potential of the proposed combination of LAPS and LAE. The flexible generation of pH gradients, using a LAE by changing the illumination spot, offers the spatially resolved control of the enzyme activity inside the microfluidic channel. Furthermore, the triggered enzymatic reaction rate can be label-free monitored by the enzyme-LAPS to validate the resulting impact on the enzymatic inhibition.

## 4. Conclusions

In this work, a LAE/microfluidic foil/LAPS sandwich structure was utilized for the detection and manipulation of pH gradients inside a microfluidic system. The LAPS offers the ability to detect spatially resolved pH changes inside the microfluidic channel. In contrast, locally induced pH changes can be triggered using the LAE, whereby the location may be controlled by the illuminated area. To study this sensing-actuating interplay, as a model bioreceptor, the enzyme penicillinase was immobilized inside the microchannel using plant viral (TMV) particles as enzyme nanocarriers. The enzymatic cleavage of penicillin to penicilloic acid, yielding H^+^ ions, leads to local pH changes, which can be detected by the LAPS. By inducing a further pH shift via the LAE, the enzymatic activity can be inhibited. 

The novel actuator-sensor platform was characterized performing photocurrent-voltage-, photocurrent-time measurements and chemical imaging with the LAPS and by transient current measurements with the LAE. The surface morphology of the LAE and TMV-modified LAPS was analyzed by means of SEM. 

In the concentration range from 0.1 to 5.0 mM penicillin, the TMV-penicillinase-modified LAPS sensor achieved a penicillin sensitivity of 42.3 mV/dec, proofing the functionality as penicillin sensor inside the microfluidic setup. Additionally, the chemical images visualize, that the TMV-immobilized enzymes were confined to the area, predefined through drop-coating during assembly of the system. This extends the use of beneficial plant viral enzyme nanocarriers to a further type of microsystem. For solutions of varying pH, the inhibition of the enzymatic reaction was demonstrated at pH 4.0, whereas enzyme activity increased in LAPS measurements when pH is shifted towards the enzyme’s pH optimum. Furthermore, a strategy for a spatially resolved photoelectrocatalytical pH manipulation induced by the LAE was developed. Such a pH gradient inside the microchannel was utilized to control the enzymatic reaction. 

By this exemplary application, the feasibility and potential of combining two light-addressable technologies, LAPS and LAE, was highlighted to be of great benefit for further integration in lab-on-a-chip systems. The advantage of this system lies in the adaptability of both technologies, as the region of interest inside, e.g., the microfluidic channel, can be regulated in time and geometrical locus by changing the illuminated area. 

In future studies, such as e.g., enzyme arrays inside a microfluidic channel, each particular enzyme might be controlled individually. Dependent on the adjusted pH value by the LAE, the enzyme activity can be either shifted to the enzyme’s pH optimum (leading to increased reaction rates) or to pH values, where inhibition of enzyme takes place.

A further interesting approach for such actuator-sensing platform lies in the field of enantioselective enzymes which catalyse multiple reactions (e.g., acetoin reductase for acetoin and diacetyl determination [34,35]). Here, local pH variations triggered by the LAE could shift the pH optima corresponding to the respective substrate molecule of interest. A separation by different microchannels will address individual enzymes having a two-dimensional monitoring of each single reaction.

## Supplementary Materials

The following are available online at https://www.mdpi.com/article/10.3390/bios11060171/s1, Figure S1: Reference chemical image of the microfluidic structure recorded at an applied potential of −1.65 V in 0.33 mM PBS buffer, pH 7.0. Figure S2: Photocurrent-time curve for 1.0 mM penicillin in PBS buffer, pH 7.1, for different flow rates.
