# Supplementary material for: Light-Addressable Actuator-Sensor Platform for Monitoring and Manipulation of pH Gradients in Microfluidics: A Case Study with the Enzyme Penicillinase
Source: Biosensors (Basel). 2021 May 27;11(6):171. doi: 10.3390/bios11060171 (PMC8230332; doi:10.3390/bios11060171)
Supplement: Supplementary file 1 [file biosensors-11-00171-s001.zip › biosensors-1223881-supplementary.pdf]

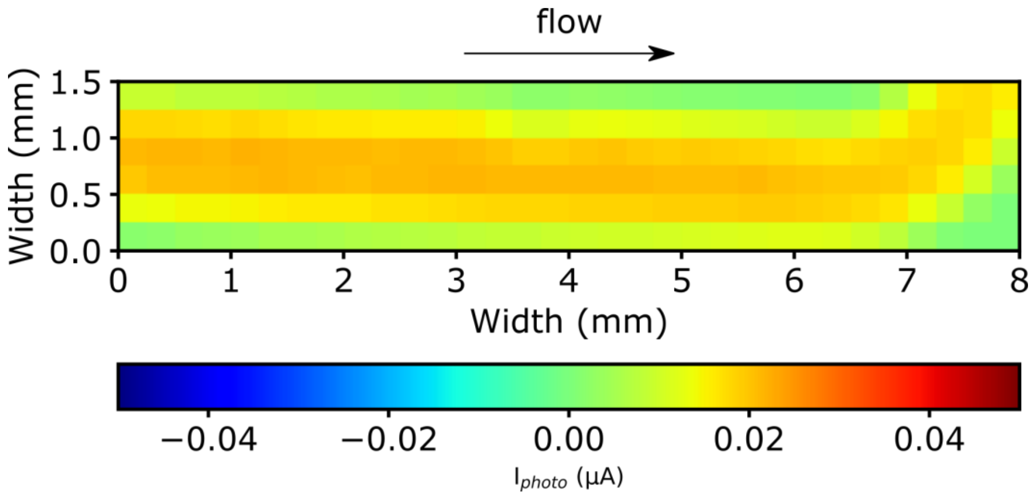

Figure S1: Reference chemical image of the microfluidic structure recorded at an applied potential of -1.65 V in 0.33 mM PBS buffer, pH 7.0.

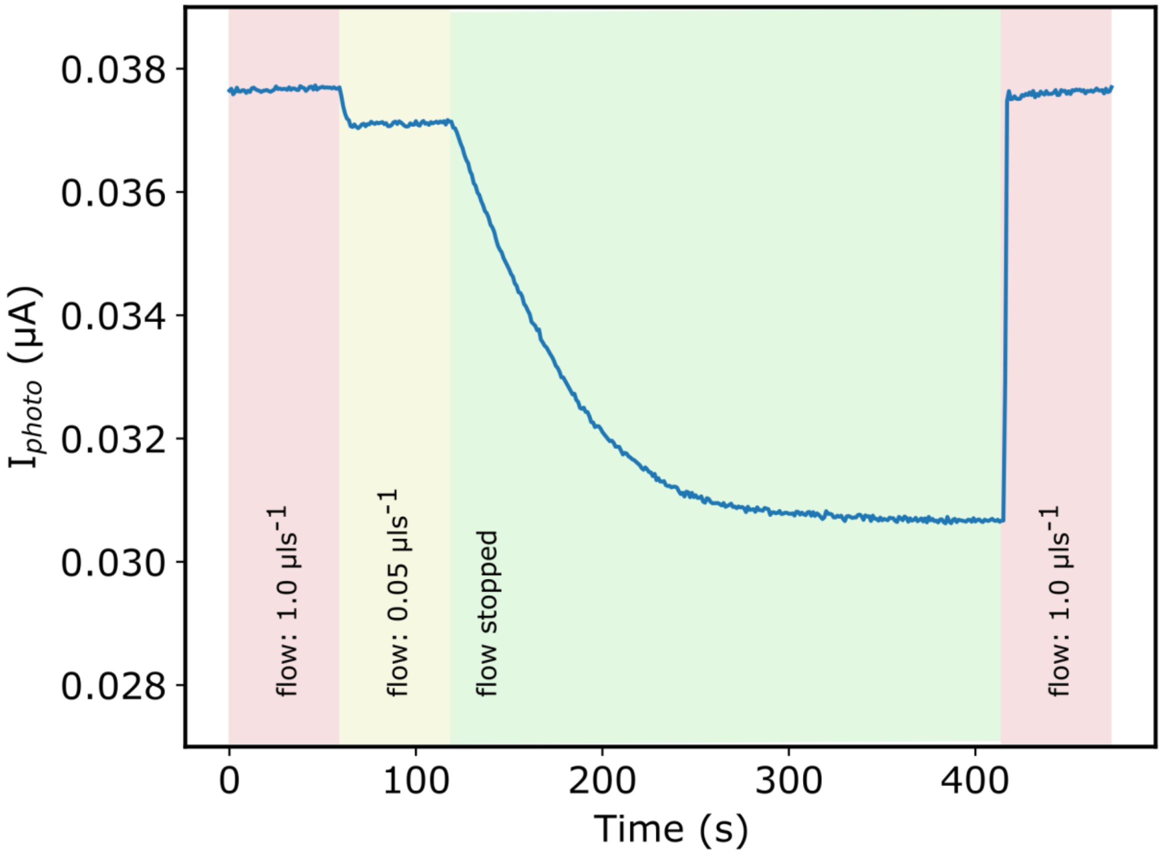

Figure S2: Photocurrent-time curve for 1.0 mM penicillin in PBS buffer, pH 7.1, for different flow rates.
